# Assessing the impact of aprepitant and ondansetron on postoperative nausea and vomiting in orthognathic surgeries: a randomized controlled trial

**DOI:** 10.1186/s12871-023-02371-y

**Published:** 2023-12-13

**Authors:** Mostafa Alam, Alireza Shakeri, Ardeshir Khorsand, Karim Nasseri, Sadaf Nasseri

**Affiliations:** 1 Department of Oral and Maxillofacial Surgery, School of Dentistry, Shahid Beheshti University of Medical Sciences, Tehran, Iran; 2 Department of Anesthesiology, Shahid Beheshti University of Medical Sciences, Tehran, Iran; 3 Department of Anesthesiology, Faculty of Medicine, Kurdistan University of Medical Sciences, Sanandaj, Iran; 4 Research Institute of Dental Sciences-Shahid Beheshti University of Medical Sciences, School of Dentistry, Shahid Beheshti University of Medical Sciences, Tehran, Iran; 5 Department of Oral and Maxillofacial Medicine, Health Service, Medical University of Kurdistan, Sanandaj, Iran

**Keywords:** Aprepitant, Ondansetron, Orthognathic surgery, Postoperative nausea and vomiting

## Abstract

**Background:**

Postoperative nausea and vomiting (PONV) is a common side effect associated with general anesthesia. Both ondansetron and aprepitant been effectively used to prevent PONV. However, there is a disagreement of opinions regarding the superiority of these two drugs. This study aims to compare the efficacy of aprepitant with ondansetron in preventing PONV following orthognathic surgeries.

**Methods:**

In this double-blinded clinical trial, 80 patients scheduled for orthognathic surgery at Imam Hossein Hospital, Tehran, Iran, were randomly assigned to two groups. A standardized anesthesia protocol was used for all patients. The first group received a placebo capsule administered one hour before the surgical procedure along with 4 mg (2 ml) of ondansetron intravenously after anesthesia induction. The second group was given 80 mg aprepitant capsules one hour before the surgery, followed by an injection of 2 ml intravenous distilled water after anesthesia induction.

The occurrence and severity of PONV, the amount of rescue medication required, and the complete response of patients assessed within 24 h after the surgery.

**Results:**

There were no significant differences in demographic data between the two groups. Patients in the aprepitant group had a significantly lower incidence and severity of nausea (2.5% versus 27.5%), vomiting (5% versus 25%), and required fewer rescue medications (7.5% versus 62.5%) compared to the ondansetron group. Additionally, the aprepitant group showed a higher complete response rate (90% versus 67.5%) in the 0-2 and 12-24 postoperative hours.

**Conclusion:**

According to the findings of this study, aprepitant has demonstrated a greater efficacy in preventing PONV following orthognathic surgery, when compared to ondansetron.

**Trial registration:**

Iranian Registry of Clinical Trials (IRCT code: IRCT20211205053279N3), date of registration: 16/12/2022.

## Introduction

Post-operative nausea and vomiting (PONV) is a common side effect of general anesthesia that can significantly decrease patient satisfaction. In fact, many patients find PONV more distressing than postoperative pain [1]. According to a systematic review, the average prevalence of PONV is around 36%, but this can increase up to 92% depending on the type of surgery, anesthesia technique, and patient's risk factors [2]. Orthognathic surgeries, in particular, have a higher incidence of PONV due to bleeding, swelling of the oral cavity, and a special diet after surgery [3]. While PONV is not typically life threatening, it can lead to dehydration, electrolyte imbalance, subcutaneous emphysema, and even esophageal rupture in severe cases. In orthognathic surgeries, it can also cause damage to the operating area, wound dehiscence, and hematoma. In cases where intermaxillary fixation is used, vomiting can lead to aspiration and airway closure [4].To prevent PONV in patients undergoing orthognathic surgery, prophylactic antiemetics are essential. There are various classes of antiemetic drugs available, including antagonists of dopamine, serotonin, neurokinin 1, histamine, and acetylcholine. Agonists of cannabinoids, corticosteroids, and benzodiazepines also have some antiemetic properties. However, the use of drugs that are antagonized the 5-hydroxytryptamine 3 (5-HT3) receptors, such as ondansetron, dolasetron, tropisetron, and ramosetron, is more common for the prevention of PONV. While these drugs cannot completely prevent the occurrence of PONV, they have a high effect and rare complications [5].

Aprepitant is a highly selective neurokinin-1 (NK1) receptor and substance P antagonist. It is commonly used to prevent nausea and vomiting in patients undergoing chemotherapy, as well as to prevent opioid-induced emesis [6, 7]. With a half-life of 9-12 h, aprepitant is highly effective in preventing both acute and delayed emesis [8]. Recent studies have shown that aprepitant is also effective in preventing PONV in cancer, abdominal, and bariatric surgeries. However, there is currently no research on its efficacy in orthognathic surgeries, which had been known to be high-risk for PONV [9–11]. The current study aims to investigate the efficacy of aprepitant compared to ondansetron in preventing PONV after orthognathic surgeries.

## Methods

### Study design ethics, and patient population

This study was a double-blinded, randomized clinical trial that was carried out in the oral and maxillofacial surgery department of Imam Hossein Hospital (Tehran-Iran). This study was approved by the Research Ethics Committees of the research institute of Dental sciences-Shahid Beheshti University of medical sciences and registered at the Iranian Registry of Clinical Trials (IRCT code: IRCT20211205053279N3) and conducted following the Declaration of Helsinki. All patients provided written informed consent to participate in the study.

All patients between the ages of 15-50 years with an American Society of Anesthesiologists (ASA) physical status of I–II scheduled to undergo orthognathic surgery under general anesthesia were enrolled in this study between April 31, 2022, and March 8, 2023. Exclusion criteria were as follows: abnormal liver or renal function, patients with known hypersensitivity to ondansetron or aprepitant, and pregnant or nursing mothers. Patient information, including gender, history of PONV and motion sickness, and smoking status was recorded. Computer-generated random numbers divided the patients into two groups. Randomization was forwarded directly to a nurse who was not involved with the patients’ management and assessment.

### Intervention

The first group received a placebo capsule administered one hour before the surgical procedure along with 4 mg (2 ml) of ondansetron intravenously after anesthesia induction. The second group was given 80 mg aprepitant capsules one hour before the surgery, followed by an injection of 2 ml intravenous distilled water after anesthesia induction.

All patients undergo Lefort I maxillary advancement osteotomy and Bilateral Sagittal Split Osteotomy mandibular setback surgery with the Dalpont method. Fixation of the maxilla was done by 4 L-shaped plates with 4 holes and fixation of the mandible by 3 screws.

The anesthesia method of all patients was similar. Firstly, initial standard monitoring including non-invasive blood pressure (NIBP), heart rate (HR), electrocardiogram (ECG), end-tidal-CO_2_ (ETCO2), and pulse oximetry (SPO2) monitoring was applied. Patients were hydrated with 5ml/kg of intravenous crystalloid fluid. Premedication with intravenous administration of 0.2 mg/kg midazolam and 2 mcg/kg fentanyl was performed 5 min before anesthesia induction. Anesthesia induced with 2 mg/kg propofol, and muscle relaxation was provided with 0.5 mg/kg atracurium, followed by 1mg/kg lidocaine 90 s before intubation. Anesthesia was maintained with a combination of oxygen and air with 1–1.5% isoflurane. At the end of the operation, 0.05mg/kg of neostigmine and 0.02mg/kg of atropine were administered to reverse residual neuromuscular block. Patients were extubated and transferred to the post-operative care unit (PACU) for further observation.

### Assessment of outcomes

The duration of surgery and the total amount of administered narcotics were recorded. All patients were kept in PACU for two hours and while assessing their vital signs (BP, HR, and SPO2), the occurrence and severity of PONV were evaluated and recorded as the primary outcome. In addition, the feeling of nausea, the frequency of vomiting, and the amount of receiving antiemetics rescue medicine after transferring to ward in time period intervals up to 24 h after the surgery were evaluated by an investigator who was unaware of the patients group. The 11-point verbal rating scale (VRS) index was used to record nausea. In this index, patients were asked to assign a number from 0 to 10 to assess their feeling of nausea, where the number zero means the absence of nausea and the number ten most intense feelings of nausea. The investigator also recorded the number of vomiting. A single dose intravenous 20 mg metoclopramide used as the rescue therapy of nausea, which allowed it to be administered by the investigator in case of vomiting; nausea with a score higher than seven did that lasted for more than 15 min and at the patient's own request.

### Statics

Based on Gan et al. [6] and assuming 20% differences between aprepitant and ondansetron in preventing PONV, for a two-sided test to compare the two proportions at 0.05 significance level, we estimated the minimum number of 39 patients in each group to achieve 80% power.

The data was analyzed using Statistical Package for Social Sciences (SPSS) software (version 21, SPSS INC., Chicago, IL, USA). Demographic and other patient characteristics were expressed as means ± standard deviations. Values < 0.05 were considered statistically significant. For analyzing patient demographics, the cumulative incidence of vomiting at each time point, the incidence of nausea, rescue antiemetic use, and complete response (no vomiting and no rescue) in periods of 0–2, 2–6, 6–12, and 12-24 h after surgery t-test and *K*2 test were used.

## Result

**Fig. 1:**
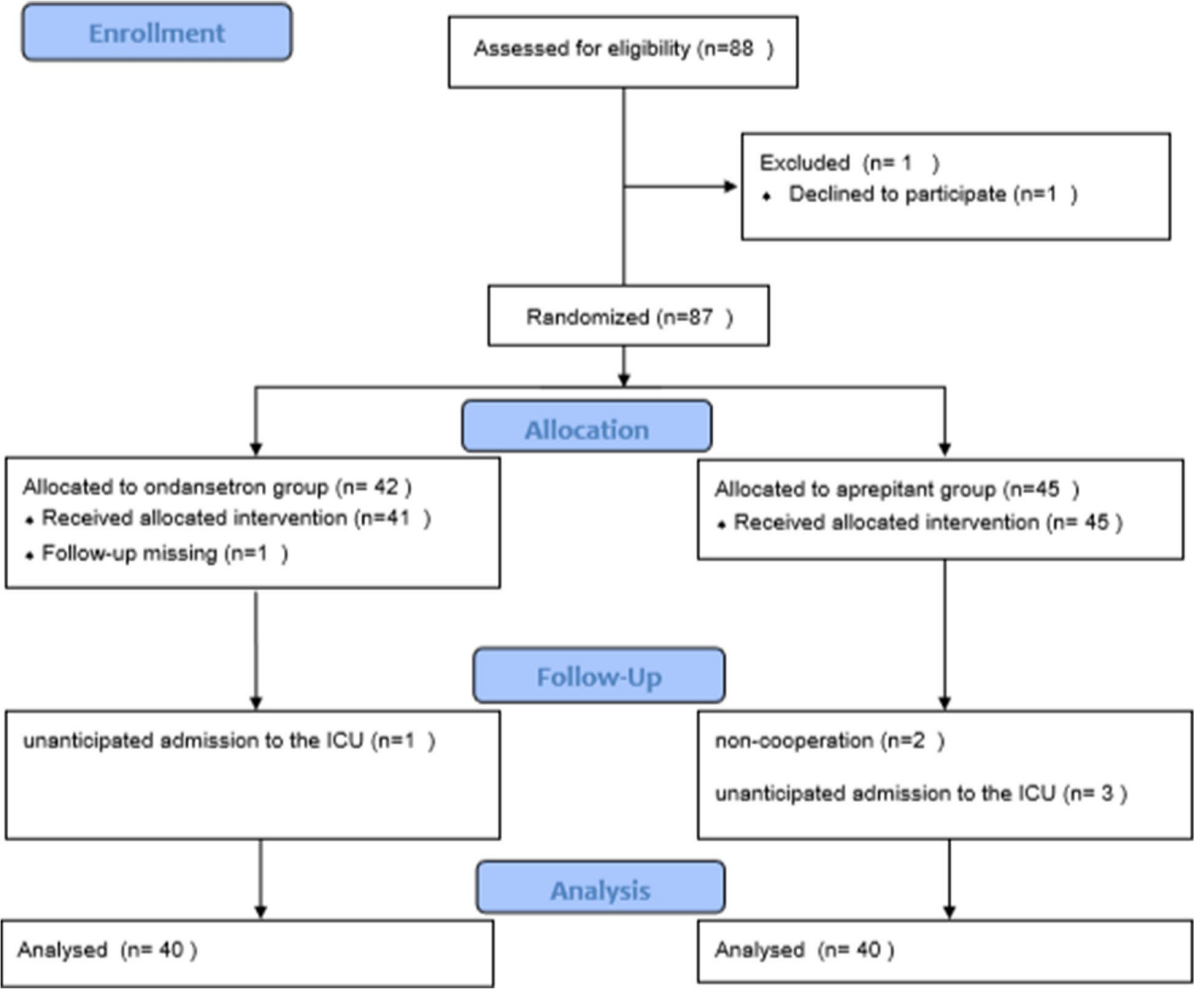
CONSORT flow diagram of participants

Eighty-eight patients were assessed for eligibility in the study. One patient withdrew their consent for participation in the study before the start of the intervention. Eighty-seven patients were randomly divided into two groups. Forty-two patients received ondansetron and 45 patients received aprepitant. Due to follow-up missing one participants from the ondansetron group were excluded from the study. Due to no extubation and unanticipated admission to the ICU one participants from the aprepitant group were excluded from the analysis. Five participants from the aprepitant group were excluded from the analysis. Two people were excluded from the analysis due to the non-cooperation in follow up, and three patients were due to no extubation and unanticipated admission to the ICU. Finally, forty people from each group were analyzed (Fig. 1).

Demographic data and risk factors were comparable and there were no significant differences between two groups. The surgery duration and the amount of opioid received by the patients during surgery were also recorded and analyzed, which were not significantly different from each other (Table 1).

**Table 1:** Background factors and those related to operation and anaesthesia

Group	Ondansetron (*n* = 40)	Aprepitant (*n* = 40)	*P*-value
Variable			
Female [n (%)]	20 (50)	20 (50)	0.87
Age [mean (SD)]	34 (10.7)	33 (11.7)	0.76
Weight [mean (SD)]	67 (18.1)	68 (13.0)	0.87
BMI [mean (SD)]	23 (3.3)	22 (3.4)	0.76
Surgery duration [mean (SD)]	3.8 (1.4)	3.7 (1.4)	0.87
ASA Physical status [n(%)]			
I	32 (80)	31 (77)	0.07
II	8 (20)	9 (23)	
Motion sickness [n (%)]	6 (15)	7 (17.5)	0.09
PONV history [n (%)]	3 (7.5)	4 (10)	0.15
Non-smocking [n (%)]	31 (77.5)	30 (75)	0.06
Amount of fentanyl (mcg) prescribed during surgery[mean (S*D)]*	211 (61)	222 (60)	0.41

*N* number, *SD* Standard Deviation, *BMI* Body Mass Index, *ASA* American Society of Anesthesiologist, *PONV* Post-Operative Nausea and Vomiting, *mcg* microgram

It was found that patients who received aprepitant had a significantly lower incidence and severity of nausea within the 0-2 h after surgery. No significant difference was observed between the two groups regarding the nausea in the time intervals of 2-6 h and 6- 12 h after surgery. However, within 12-24 h after surgery, patients of aprepitant group experienced significantly less nausea (Table 2).

**Table 2:** Amount of nausea and nausea VRS

Variable	0–2 h		*P*-value	2–6 h		*P*-value	6–12 h		*P*-value	12–24 h		*P*-value
	Group O	Group A		Group O	Group A		Group O	Group A		Group O	Group A	
Nausea (n)	11	1	0.002	6	4	0.36	5	1	0.10			0.05
Nausea VRS			0.002			0.46			0.09			0.04
0-3	30	39		34	36		36	39		37	40	
4-6	4	0		2	2		2	1		2	0	
7-10	6	1		4	2		2	0		1	0	

*n* number, *O* Ondansetron, *A* Aprepitant, *VRS* Verbal Rating Scale

The incidence of vomiting was significantly lower in the aprepitant group compared to the ondansetron group (5% versus 25%). Additionally, in the ondansetron group, a significantly higher number of patients requested rescue drugs compared to the aprepitant group (*p* < 0.05). Furthermore, the aprepitant group showed a significantly higher rate of complete response to the study drugs compared to the ondansetron group (*p* < 0.05) (Table 3).

**Table 3:** Number of patients who had vomiting, Request for rescue drug, and with complete response

Group	Ondansetron	Aprepitant	*P*-value
Variable			
Vomiting (n)	10	2	0.01
Request for rescue drug (n)	12	3	0.01
Complete Response(n)	28	37	0.01

*n* number

The most frequently reported side effect was drowsiness. Additionally, one patient in the aprepitant group experienced abdominal pain. The side effects caused by the drugs were the same between the two groups (Table 4).

**Table 4:** Complication of drugs between two groups

Complication	Group O	Group A	*p*-Value
Vertigo (n)	13	11	0.626
Blurred vison (n)	8	3	0.105
Head ache (n)	13	12	0.809
Drowsiness (n)	18	19	0.823

*n* number, *O* Ondansetron, *A* Aprepitant

## Discussion

Advancements in surgical techniques and the development of new anesthesia drugs have significantly reduced the occurrence of severe, life-threatening side effects. However, despite these improvements, the issue of PONV remains a persistent concern and none of the current antiemetic drugs has been able to resolve this simple but important problem.

Apipan et al. consider that PONV is the most common complication after orthognathic surgery under general anesthesia. This complication causes a decaying and undesirable feeling, and the occurrence of serious but albeit complications such as aspiration, postoperative hypoxemia, water and electrolyte disturbances, opening of the surgical incision, and delay in the discharge of patients [12]. Various factors such as age, gender, previous history of PONV, motion sickness, type of surgery, duration of anesthesia and surgery, and anxiety of the patient are influential factors in causing nausea and vomiting that are not controlled by the attending physician. Several methods and drugs are used to treat this complication, including metoclopramide, droperidol, propofol, and dexamethasone. The research on reducing PONV focuses on practical methods and drugs with low complications. It is obvious that the best medicine is the one that has the greatest effect and time of effect and the least side effect. In addition, it is cheap. The aim of the current study was to compare the effect of aprepitant and ondansetron on nausea and vomiting after orthognathic surgery.

In a double-blind clinical trial conducted by Salome Jeyabalan et al., the researchers aimed to compare the efficacy of two different treatment regimens in preventing PONV. They divided 120 patients who underwent abdominal or thyroid surgery into two groups. One group received ondansetron along with aprepitant, while the other group received ondansetron with a placebo. The primary outcomes measured were the incidence of nausea and vomiting, as well as the time required for rescue drugs to be administered. They concluded that a single dose of 40 mg of aprepitant had a comparable effect to 8 mg of intravenous ondansetron every 8 h in preventing PONV, the severity of nausea, the number of times rescue medication was requested, and the time of the first nausea attack in 24 h after surgery. The findings of this study about the effectiveness of aprepitant on PONV are similar to our study. However, the design of their study is different from our study because they injected ondansetron in both groups at the end of the operation and then every 8 h, while in our study only one group of patients received ondansetron and the patients in the aprepitant group received distilled water instead of ondansetron [13]. Comparablelity of 40 mg of aprepitant to multiple doses of intravenous ondansetron suggests that aprepitant may be a more convenient and cost-effective option for preventing PONV. That, potentially reducing the overall medication burden and associated costs.

In a randomized double-blind study, Diemensch P et al. compared the effect of two single doses of aprepitant (40 mg and 125 mg) with 4 mg of intravenous ondansetron on the prevention of PONV in 922 patients who underwent general anesthesia for open abdominal surgery. They recorded episodes of vomiting, the use of rescue medication, and the severity of nausea for 48 h. The primary outcome was a complete response (no vomiting and no use of rescue therapy) within 0-24 h of surgery. They concluded that both doses of aprepitant were non-inferior to ondansetron in achieving a complete response in the first 24 h. Aprepitant was significantly more effective than ondansetron in preventing vomiting and reducing nausea in both 24 and 48 h. The findings of our study are consistent with the mentioned study [9].

In a randomized double-blind study, Bergese et al. compared the three drugs of aprepitant, dexamethasone, and promethazine with ondansetron, dexamethasone, and promethazine on declining the incidence of PONV in 176 patients who underwent craniotomy under general anesthesia. The researchers concluded that both triple-drug regimens have a similar effect on the prevention of PONV. The results of this study regarding the superiority of ondansetron over aprepitant are different from our study. The explanation for the discrepancy in the results between our study and the study conducted by Bergese et al. is not entirely clear. However, the differences in patient populations between the two studies, variations in the dosages or administration of two additional drugs (dexamethasone, and promethazine), and differences in study design (sample size, randomization methods, blinding techniques, and outcome measures), may contribute to discrepancies between two studies [14].

Gan TJ et al. in a multicenter study compared the effect of two different doses of oral aprepitant for the prevention of PONV on patients who underwent open abdominal surgery under general anesthesia. In their study, patients divided into three groups. The first and second groups received 40 and 125 mg of oral aprepitant, respectively, and the third group received 4 mg of intravenous ondansetron. The researchers concluded that for the prevention of vomiting in the first 48 h after the operation, aprepitant is superior to ondansetron, but there is no significant difference between aprepitant and ondansetron in controlling nausea, using rescue drugs, and complete response. The results of our study regarding the occurrence of vomiting are consistent with the results of the above study. However, regarding the use of rescue drugs and control of nausea and complete response, the results of this study are inconsistent with our study, which be attributed to the difference in kind of surgery in the patients of the two studies and the possibility of lower chance of complete response and reduction of nausea in open abdominal surgeries [6].

In a randomized double-blind clinical trial, Hassan AME et al. divided 150 patients who underwent laparoscopic bariatric surgery into three groups (50 patients in the ondansetron group (A), 50 patients in the aprepitant group (B), and 50 patients in the ondansetron group + aprepitant [15]. They injected intravenous dexamethasone into all patients. The primary outcome of the study was the severity of nausea with a complete response in the first 48 h after the operation. The researchers concluded that adding aprepitant to ondansetron significantly reduces the amount of vomiting and the severity of nausea and reduces the complete response to 48 h compared to ondansetron. Furthermore, oral aprepitant in combination with intravenous ondansetron and dexamethasone is effective in suppressing primary PONV up to 48 h after surgery. The results of this study are consistent with our study [15].

Safarnejad F et al. compared the effect of aprepitant and ondansetron on PONV following laparoscopic cholecystectomy separately and in combination in their study. They divided patients into three groups. The first group received 80 mg of oral aprepitant, the second group received 4 mg of intravenous ondansetron, and the third group received 4 mg of intravenous ondansetron and 80 mg of oral aprepitant. Nausea and vomiting in the third group were less than the other two groups. In addition, the occurrence of vomiting in the first group was significantly lower than in the second group. Results of the mentioned study were consistent with our findings [16].

In a systematic review, Liu et al. reviewed 14 clinical trials about the effects of two doses of 40- and 80-mg of aprepitant on PNOV. According to their systematic review, both doses of aprepitant are more effective than 4 mg of ondansetron in preventing PONV. According to the results of the mentioned studies and the current study, in the first 24 h after the surgery, aprepitant compared to ondansetron significantly reduces the incidence and severity of nausea and vomiting, use of rescue medication, and increases complete response [17].

Kakuta and colleagues in a study investigated the effectiveness of aprepitant on nausea and vomiting, and post-operative pain. They concluded that aprepitant not only reduces nausea and vomiting but also increases pain tolerance. Investigation about postoperative pain was not among our study goals [18].

Aprepitant is traditionally used in the treatment of nausea and vomiting caused by cancer chemotherapy. Recently, it has been observed that aprepitant is effective not only in the treatment of PONV but also in reducing postoperative pain. Substance P is one of the neurotransmitters that is found in both central and peripheral nerves. It has been found that after binding to neurokinin 1 receptors, substance P regulates many biological functions of the central nervous system, such as emotions, negative behaviors, stress, depression, pain, and anxiety. Neurokinin 1 receptor antagonists specifically inhibit some biological functions that mediated by the binding of substance P to neurokinin 1 receptors [19].

### Limitation

The current study had several limitations. First, the aprepitant was administered 60 min before the induction of anesthesia. Considering that it takes approximately 3 h for the oral aprepitant to reach the maximum blood concentration [20], it is possible that in some patients who had a short surgery period, the aprepitant did not reach its maximum blood concentration at the end of the surgery, which can affect the results. Though this induces a negative effect on the aprepitant group, the effectiveness of the aprepitant was higher than ondansetron. Secondly, both propofol and midazolam, which may affect the incidence of PONV, were used to induce anesthesia in this study. However, the use of similar doses of propofol and midazolam for induction of anesthesia in both groups neutralizes the bias effect caused by the use of these drugs. Finally, although the sample size was calculated based on previous studies, it seems that the use of a higher sample size would have led to more confidence in the results.

## Conclusion

According to the findings of this study, aprepitant has demonstrated a greater efficacy in preventing postoperative nausea and vomiting following orthognathic surgery, when compared to ondansetron.

## Data Availability

The datasets used and/or analyzed during the current study are available from the corresponding author on reasonable request.

## Abbreviations

PONV: Post-operative nausea and vomiting
NK1: Neurokinin-1
IRCT: Iranian Registry of Clinical Trials
ASA: American Society of Anesthesiologists
NIBP: Non-invasive blood pressure
HR: Heart rate
ECG: Electrocardiogram
ETCO2: End-tidal-CO2
SPO2: Pulse oximetry
5-HT3: 5-Hydroxytryptamine 3
PACU: Post-operative care unit
VRS: Verbal rating scale
SPSS: Statistical Package for Social Sciences
